# Obesity, hypertension and diuretic use as risk factors for incident gout: a systematic review and meta-analysis of cohort studies

**DOI:** 10.1186/s13075-018-1612-1

**Published:** 2018-07-05

**Authors:** Peter L. Evans, James A. Prior, John Belcher, Christian D. Mallen, Charles A. Hay, Edward Roddy

**Affiliations:** Research Institute for Primary Care and Health Sciences, Keele University, Staffordshire, ST5 5BG UK

**Keywords:** Gout, Systematic review, Meta-analysis, Rheumatology

## Abstract

**Background:**

Gout treatment remains suboptimal. Identifying populations at risk of developing gout may provide opportunities for prevention. Our aim was to assess the risk of incident gout associated with obesity, hypertension and diuretic use.

**Methods:**

We conducted a systematic review and meta-analysis of prospective and retrospective cohort studies in adults (age ≥ 18 years) from primary care or the general population, exposed to obesity, hypertension or diuretic use and with incident gout as their outcome.

**Results:**

A total of 9923 articles were identified: 14 met the inclusion criteria, 11 of which contained data suitable for pooling in the meta-analysis. Four articles were identified for obesity, 10 for hypertension and six for diuretic use, with four, nine and three articles included respectively for each meta-analysis. Gout was 2.24 times more likely to occur in individuals with body mass index ≥ 30 kg/m^2^ (adjusted relative risk 2.24 (95% confidence interval) 1.76–2.86). Hypertensive individuals were 1.64 (1.34–2.01) and 2.11 (1.64–2.72) times more likely to develop gout as normotensive individuals (adjusted hazard ratio and relative risk respectively). Diuretic use was associated with almost 2.5 times the risk of developing gout compared to no diuretic use (adjusted relative risk 2.39 (1.57–3.65)).

**Conclusions:**

Obesity, hypertension and diuretic use are risk factors for incident gout, each more than doubling the risk compared to those without these risk factors. Patients with these risk factors should be recognised by clinicians as being at greater risk of developing gout and provided with appropriate management and treatment options.

## Background

Gout affects 2.5% of adults in the UK, with prevalence and incidence continuing to rise [1, 2]. The primary risk factor for gout is an elevated serum urate level (hyperuricaemia), leading to monosodium urate crystal deposition in and around joints, acute attacks of crystal synovitis and progressive joint damage [3]. Long-term treatment of gout involves using urate-lowering therapies (ULT), typically allopurinol [4] to inhibit xanthine oxidase, resulting in improved long-term outcomes. Despite this, treatment use remains suboptimal [5] and, therefore, identifying populations at risk of developing gout, especially those in primary care where the majority of patients with gout are managed, may provide opportunities for primary prevention.

Body mass index (BMI) and hypertension have been identified as risk factors for incident gout in a number of large epidemiological studies [6], yet the magnitude of risk varies between studies. Obesity promotes insulin resistance which in turn reduces renal urate excretion resulting in hyperuricaemia [7]. Hypertension predisposes to gout by reducing renal urate excretion due to glomerular arteriolar damage and glomerulosclerosis. Diuretics are perhaps the most well-known medications to be associated with gout; they raise serum uric acid levels by increasing uric acid reabsorption and decreasing uric acid secretion in the kidneys. However, it has also been proposed that diuretic use alone does not increase the risk of gout and that the observed associated risk is due to the presence of co-morbidities which they are used to treat; commonly hypertension, heart failure and renal failure [8]. Studying obesity, hypertension and diuretic use and their association with incident gout to elucidate the true nature and magnitude is important because all three are common and can be modified. We performed a systematic review of cohort studies with the aim of deriving pooled estimates of the risk of incident gout associated with obesity, hypertension and diuretic use.

## Methods

### Literature search

We searched MEDLINE, Embase, CINAHL and the Cochrane Library from their inception to March 2017. A combination of free-text and medical subject heading (MeSH) terms, or database-specific equivalents, were used (Appendix 1). Reference lists of included articles were searched for additional eligible articles.

### Inclusion and exclusion criteria

The inclusion criteria were developed using the PICOS framework [9]. The population of interest was adults aged 18 years or older. Studies which included participants under the age of 18 years at cohort entry, but in whom outcome was assessed in adulthood, were deemed to meet this inclusion criterion. Articles were required to have examined at least one of: obesity (BMI ≥ 30 kg/m^2^), hypertension (self-reported, physician-diagnosed or study-defined mmHg value) or diuretic use (self-reported or reported in records) and their association with incident gout, defined as the first recorded episode (i.e. a subsequent new diagnosis of gout). Articles which studied the incidence of gout in specific hyperuricaemic populations were excluded. Included articles had to be cohort studies, prospective or retrospective and undertaken in primary care or the general population.

No restrictions were imposed on language or the time periods for publication, with medical literature databases searched from inception. Where full articles could not be obtained, these were requested from the corresponding author.

### Screening process

After duplicates had been removed from the initial search, the titles and abstracts of all of the remaining articles were screened by two authors (PLE and CH). Two authors (PLE/CH and JAP) then independently reviewed the full text of the remaining articles to decide on inclusion. Any articles where there was disagreement about inclusion were subsequently arbitrated over by a third author (ER).

### Data extraction and quality assessment

Data were extracted from the full set of eligible articles by a single author (PLE/CH) and also extracted independently from a subset (50%) of the eligible articles by a second author (JAP). If risk estimates were not reported in the original articles, these were requested from the corresponding author. Extracted variables included author, year and title of publication, country in which the study took place, the number of years of follow-up, baseline demographics of participants which included age, gender and ethnicity, the number of cases of incident gout, the study setting (primary care or general population), exposure of interest and method of definition used, the method of gout diagnosis and the risk estimate of incident gout associated with that particular exposure, and both unadjusted and adjusted values were extracted if available. The methodological quality of all eligible articles was assessed independently by two authors (PLE/CH and JAP) using the cohort study template of the Newcastle–Ottawa Scale (NOS). Funnel plots were produced from the adjusted data points for each meta-analysis to examine the extent of any publication bias.

### Statistical analysis

Narrative synthesis was used to summarise the characteristics of studies included in the systematic review. Estimates were pooled for an individual risk factor if there were a minimum of three values which met the criteria; the exposure was measured in a similar manner and used the same risk estimate (e.g. odds ratio (OR), relative risk (RR), hazards ratio (HR)). Firstly, unadjusted or minimal adjusted risk estimates were pooled for each individual risk factor, and then the maximal multivariate-adjusted risk estimates.

Pooled risk estimates were calculated using random-effects meta-analysis. A random-effects model is considered more appropriate for meta-analyses with the potential for substantial heterogeneity. Quantifying the inconsistency across studies was assessed using Cochran *Q* and *I*^2^ statistics. The DerSimonian and Laird random-effects models were then used to calculate the pooled risk together with associated 95% confidence intervals (CI). The meta-analysis was performed using STATA 13.

## Results

### Search results

**Fig. 1:**
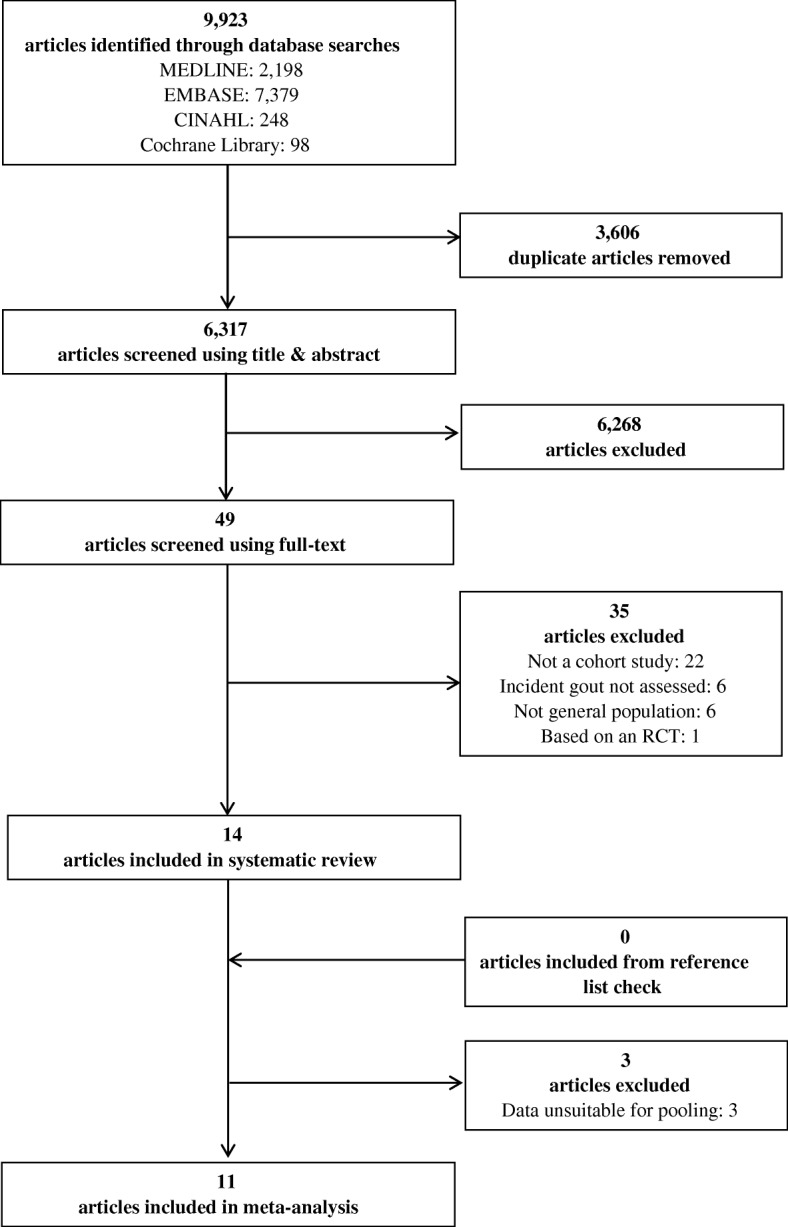
Number of articles at each stage of the search and screening process

The search yielded a total of 9923 articles. Of these 3606 were duplicates and hence removed, leaving 6317 individual publications to be screened by title and abstract. Forty-nine articles could not be excluded by title and abstract and had their full text reviewed. Thirty-five articles were excluded (Appendix 2), 14 articles met the inclusion criteria [10–23], no additional articles were identified in their reference lists and a final 11 articles contained data suitable for pooling in the meta-analysis [11, 13–18, 22–25] (Fig. 1).

### Characteristics of included articles

Thirteen of the 14 articles included in the systematic review used general populations, with one from primary care [13] (Table 1). The majority were from the USA, with the remaining four articles from the UK, Singapore [22], Taiwan [18] and Tokelau (South Pacific island) [10]. Sample sizes for the included articles ranged from 923 to 60,181, with the number of incident cases of gout ranging from 43 to 1341. Two studies included only male participants—one using a cohort of male health professionals [14] and the other a sample of male medical students [12]—and one study included an all-female sample from the Atherosclerosis Risk in Communities (ARIC) study [17]. The remaining 11 studies included both men and women, although the sample in one study was predominantly male (91%) [11]. There was a majority of white participants (ranging from 62 to 100%) in the nine articles which reported the ethnic composition of their samples, with the exception of Prior et al. [10] which examined 100% Tokelauan. Two studies included participants who were aged under 18 years at study entry [10, 16], two studies included those in their mid-twenties (university students) [11, 12], seven studies included participants in middle-older age [14, 15, 17, 18, 20, 22, 25], one study included participants in older age [23] and two studies included wide age ranges (18–89 years) [13, 21]. The length of follow-up of participants ranged from 8 to 52 years.

**Table 1:** Characteristics of the included articles (*n* = 14)

Article	Country	Study setting (study name)	Age (years)	Gender	Ethnicity	Years of follow-up	Ascertainment of exposure	Ascertainment of gout diagnosis
Prior et al. 1987 [10]	New Zealand and Tokelau	Population based	≥ 15 at baseline, ≥ 18 at first follow-up	Men and women included, but numbers not specified	100% Tokelauan	Up to 14	Hypertension: measurement of systolic and diastolic blood pressure	History of ≥ 2 episodes of podagra with redness and swelling of first metatarsophalangeal joint
Roubenoff et al. (1991) [11]^a^	USA	Population based (medical students) (John Hopkins precursor study)	Median 22	Men: 1216 (91%); women: 121 (9%)	White: 1301 (97%); non-white: 36 (3%)	40	Hypertension: self-reported SBP > 160 mmHg or DBP > 95 mmHg on two questionnaires or self-reported anti-hypertensive medication use	Self-report followed by medical chart review
Hochberg et al. (1995) [12]^a^	USA	Population based (medical students) (John Hopkins precursor study)	White: mean 26.1, SD 1.8; black: mean 29.0, SD 3.8	Men: 923 (100%)	White: 571 (62%); black: 352 (38%)	26–34; mean 29	Hypertension: self-reported SBP > 160 mmHg or DBP > 95 mmHg on two questionnaires or self-reported anti-hypertensive medication use	Self-report plus one of: history of MSU crystals or documented tophus or use of colchicine, probenecid or allopurinol
Grodzicki et al. (1997) [13]^a^	UK	Primary care (general practice hypertension study)	18–65	Men: 1060 (50%); women: 1068 (50%)	Not reported	Average 8	Hypertension: not reported Diuretic use: not reported	Diagnosed by GP
Choi et al. (2005) [14]^a^	USA	Population based (male healthcare professionals) (health professionals follow-up study)	40–75, mean 54	Men: 47,150 (100%)	91% white	12	Obesity: self-reportedHypertension: self-reported physician-diagnosed hypertension Diuretic use: self-reported	Self-report followed by ACR criteria (≥ 6/11 for diagnosis of gout)
Bhole et al. (2010) [15]^a^	USA	Population based (Framington heart study)	Men: mean 46, SD 9; women: mean 47, SD 9	Men: 1951 (44%); women: 2476 (56%)	Not reported	52; median 28	Obesity: measured height and weight, BMI calculatedHypertension: average of two readings SBP ≥ 140 mmHg or DBP ≥ 90 mmHgDiuretic use: self-reported	Clinical diagnosis at any follow-up study examination
McAdams DeMarco et al. (2011) [16]^a^	USA	Population based (CLUE II study)	13–87 at baseline, ≥ 24 at first follow-up	Men: 6100 (39%); women: 9433 (61%)	White: 15,533 (100%)	18	Obesity: self-reported	Self-report
Maynard et al. (2012) [17]^a^	USA	Population based (ARIC)	45–64	Women: 6263 (100%)	White: 4676 (75%); black: 1587 (25%)	9	Obesity: self-reported	Self-report
Chen et al. (2012) [18]^a^	Taiwan	Population based (health insurance database)	Men: mean 46, SD 9; women: mean 47, SD 9	Men: 60,181 (45%); women: 72,375 (55%)	–	Median 7.31	Hypertension: record linkage	Record linkage: diagnostic code of gout from ICD-9 + 2× prescriptions of colchicine + prescription of urate-lowering drugs
McAdams-DeMarco et al. (2012) [25]^a^	USA	Population based (ARIC)	45–64; mean 54, SD 5.7	Men: 4709 (43%); women: 6163 (57%)	White: 8538 (79%); black: 2334 (21%)	9	Hypertension: self-report of anti-hypertension medications or measured high blood pressure	Self-report
McAdams DeMarco et al. (2012) [20]	USA	Population based (ARIC)	45–64; mean 54, SD 5.7	Men: 2445 (42%); women: 3344 (58%)	White: 3998 (69%); black: 1791 (31%)	9	Diuretic use: self-report	Self-report
Wilson et al. (2014) [21]	USA	Population based (health insurance database)	18–89	Men: 1449 (48%); women: 1584 (52%)	–	Up to 12	Diuretics: record linkage, chlorthalidone vs hydrochlorothiazide	Record linkage: ICD-9 for gout or allopurinol, febuxostat, colchicine, probenecid
Pan et al. (2015) [22]^a^	Singapore	Population based (Singapore Chinese health study)	Hyp. 61.3 (median); no Hyp. 59.3 (median)	Hyp. men: 4403 (40.7%); no Hyp. men: 7982 (40.4)	–	12	Hypertension: self-report at recruitment interview	Self-report and clinical verification
Burke et al. (2016) [23]^a^	USA	Population based (ARIC)	≥ 65	No gout (*n* = 6535): men 43.4%; gout (*n* = 230): men 52.2%	White: 100%	25	Hypertension: SBP ≥ 140 mmHg or DBP ≥ 90 mmHg, or use of a medication to treat hypertension Diuretic use: self-report of medication use	Self-report

*ARIC* Atherosclerosis Risk In Communities, *ACR* American College of Rheumatology, *BMI* body mass index, CLUE (Give us a Clue to Cancer) II study, *DBP* diastolic blood pressure, *GP* general practitioner, *Hyp.* hypertension, *ICD-9* International Classification of Diseases, ninth revision, *MSU* monosodium urate, *SBP* systolic blood pressure, *SD* standard deviation

^a^Included in meta-analysis (*n* = 7)

From the 14 articles included in this review, 11 were used within the meta-analysis. Seven articles were pooled as each had recorded a risk estimate for at least one of the risk factors of interest using RR (95% CI) and four articles were pooled based on their use of HRs (95% CI). The risk estimates from the remaining three articles were not sufficient to pool risk estimates (Table 2).

**Table 2:** Risk estimates reported by included articles (*n* = 14)

Risk factor	Author and year	Sample size	Cases of incident gout	Outcome measure	Exposure	Risk estimate	
						Minimal adjustment model	Maximal adjustment model
Obesity	Choi et al. (2005) [14]^a^	47,150	730	RR (95% CI)	BMI ≥ 30 kg/m^2^ at age 21	2.14 (1.37–3.32)^b^	1.66 (1.06–2.60)^1^
	Bhole et al. (2010) [15]^a^	1951	200	RR (95% CI)	BMI ≥ 30 kg/m^2^ in men	3.50 (2.30–5.32)^b^	2.90 (1.89–4.44)^2^
		2476	104		BMI ≥ 30 kg/m^2^ in women	3.52 (2.16–5.72)^b^	2.74 (1.65–4.58)^2^
	McAdams-DeMarco et al. (2011) [16]^a^	15,533	517	RR (95% CI)	BMI ≥ 30 kg/m^2^ at age 21	2.06 (1.38–3.07)^c^	1.82 (1.21–2.73)^3^
	Maynard et al. (2012) [17]^a^	6263	106	RR (95% CI)	BMI ≥ 30 kg/m^2^ at age 25	4.30 (2.14–8.64)^b^	2.84 (1.33–6.09)^4^
Hypertension	Prior et al. 1987 [10]	1705	46	OR (95% CI)	Systolic blood pressure	0.03 (0.02–0.05)	–
					Diastolic blood pressure	0.05 (0.03–0.07)	–
	Roubenoff et al. (1991) [11]^a^	1271	60	RR (95% CI)	Hypertension	2.70 (1.45–5.13)	–
	Hochberg et al. (1995) [12]^a^	923	60	RR (95% CI)	Hypertension (incident)	3.78 (2.18–6.58)	3.20 (1.80–5.68)^5^
	Grodzicki et al. (1997) [13]^a^	2128	45	RR (95% CI)	Hypertension	3.93 (1.60–9.70)	–
	Choi et al. (2005) [14]^a^	47,150	730	RR (95% CI)	Hypertension	3.07 (2.64–3.56)^b^	2.31 (1.96–2.72)^6^
	Bhole et al. (2010) [15]^a^	1951	200	RR (95% CI)	Hypertension—men	2.39 (1.73–3.29)^b^	1.59 (1.12–2.24)^7^
		2476	104		Hypertension—women	2.91 (1.74–4.88)^b^	1.82 (1.06–3.14)^7^
	Chen et al. (2012) [18]^a^	60,181	1341	HR (95% CI)	Hypertension—men	1.74 (1.54–1.95)^b^	1.32 (1.17–1.48)^8^
		72,375	265		Hypertension—women	2.11 (1.59–2.79)^b^	1.34 (1.02–1.77)^8^
	McAdams-DeMarco et al. (2012) [25]^a^	10,872	274	HR (95% CI)	Hypertension (time-varying)	2.87 (2.24–3.78)	2.00 (1.54–2.61)^9^
	Pan et al. (2015) [22]^a^	31,137	163 201	HR (95% CI)	Hypertension—men Hypertension—women	- -	1.67 (1.33–2.09)^10^ 2.08 (1.66–2.60)^10^
	Burke et al. (2016) [23]^a^	2956 3809	120 110	HR (95% CI)	Hypertension—men Hypertension—women	1.33 (0.84–2.09) 1.64 (1.02–2.64)	- -
Diuretic use	Grodzicki et al. (1997) [13]^a^	2128	45	RR (95% CI)	Diuretic use (and raised diastolic blood pressure)	6.25 (2.40–16.70)	–
	Choi et al. (2005) [14]^a^	47,150	730	RR (95% CI)	Diuretic use	3.37 (2.75–4.12)^b^	1.77 (1.42–2.20)^11^
	Bhole et al. (2010) [15]^a^	4427	304	RR (95% CI)	Diuretic use in men	4.31 (3.06–6.08)^b^	3.41 (2.38–4.89)^12^
					Diuretic use in women	3.23 (2.13–4.91)	2.39 (1.53–3.74)^12^
	McAdams-DeMarco et al. (2012) [20]	5789	225	HR (95% CI)	Diuretic use	1.72 (1.32–2.25)	1.48 (1.11–1.98)^13^
	Wilson et al. (2014) [21]	3033	43	Mean number of days until incident gout (SD, range)	Chlorthalidone (CTD) vs hydrochlorothiazide (HCTZ)	CTD: 183.6 (105.44, 21–362); HCTZ: 152.7 (107.60, 22–345)	–
	Burke et al. (2016) [23]	2956 3809	120 110	HR (95% CI)	Diuretic use in men Diuretic use in women	1.58 (0.89–2.81) 1.83 (1.12–2.98)	- -

*BMI* body mass index, *CI* confidence interval, *RR* relative risk, *OR* odds ratio, *HR* hazard ratio, *SD* standard deviation

^a^Included in meta-analysis (*n* = 11)

^b^Age-adjusted model

^c^Age and sex-adjusted model

^1–13^Maximal adjustment model outlined for each article in each risk factor (adjustment for other risk factor of interest highlighted in italics) as follows:

Maximal adjustment models within obesity articles:

^1^Age, total energy intake, *diuretic use*, history of *hypertension*, presence of chronic renal failure, meat intake, seafood intake, purine-rich vegetable intake, dairy food intake, alcohol intake, meat intake and fluid intake

^2^Age, education level, alcohol consumption, *hypertension*, *diuretic use*, blood glucose level, cholesterol levels and menopausal status (women only)

^3^Age, sex, alcohol intake, blood pressure, cholesterol and treatment for *hypertension* and hypercholesterolaemia

^4^Age, menopausal status, race, diabetes mellitus, *hypertension*, *diuretic use*, alcohol intake, organ meat intake and estimated glomerular filtration rate

Maximal adjustment model within hypertension articles:

^5^Ethnicity and *BMI*

^6^Age, total energy intake, *diuretic use*, *BMI*, presence of chronic renal failure, meat intake, seafood intake, purine-rich vegetable intake, dairy food intake, alcohol intake, meat intake and fluid intake

^7^Age, education level, alcohol consumption, *diuretic use*, blood glucose level, cholesterol levels and menopausal status (women only)

^8^Age, *obesity* (BMI ≥ 27 kg/m^2^), hyperlipidaemia, diabetes mellitus, alcohol drinking and cigarette smoking

^9^Sex, race, *BMI*, alcohol intake and categorical estimated glomerular filtration rate

^10^Age, sex, dialect, year of interview, educational level, *BMI***,** physical activity, smoking status, alcohol use and history of diabetes at follow-up I

Maximal adjustment models within diuretic use articles:

^11^Age, total energy intake, *BMI*, history of *hypertension*, presence of chronic renal failure, meat intake, seafood intake, purine-rich vegetable intake, dairy food intake, alcohol intake, meat intake and fluid intake

^12^Age, education level, *BMI*, alcohol consumption, *hypertension*, blood glucose level, cholesterol levels and menopausal status (women only)

^13^Sex, race, baseline *BMI*, categorical glomerular filtration rate and time-varying *blood pressure*

The covariates which each article included in its maximal adjustment model are described in detail in Table 2; however, the majority of articles adjusted for age, gender, co-morbidities, alcohol intake and food/energy intake. Several adjustment models included specific covariates; however, each of the three risk factors of interest was typically adjusted for both of the other two risk factors. Therefore, of the four obesity articles, all adjusted for hypertension and three adjusted for diuretic use; of the six hypertension articles, five adjusted for BMI and two adjusted for diuretic use; and finally, all three of the articles examining diuretic use as a risk factor for gout adjusted for hypertension and BMI.

### Quality appraisal of articles included in the meta-analysis

Three of the 11 studies (27%) included a specific sample (male healthcare professionals (*n* = 1), medical students (*n* = 2)) [11, 12, 14]. Six articles (55%) ascertained exposure using a secure record or structured interview, ranking as the highest quality approach [11, 13, 15, 17, 22, 23]. Eight articles (73%) specifically mentioned that they had excluded participants with prevalent gout at the beginning of the study [14–18, 22, 23, 25]. No studies required the diagnosis of gout to be crystal proven, two studies (18%) defined gout through clinical diagnosis [13, 15] and, although the remaining nine studies had defined a new gout diagnosis through self-report, four of these additionally used the ACR criteria [14] or a review of the medical records [11, 12, 22] to support the definition. Three articles (27%) provided unadjusted risk estimates only [11, 13, 23], with the remaining articles providing results with adjustment for at least two confounding factors. Eight articles (73%) provided a description of those lost to follow-up [11, 12, 14–17, 22, 23] (Appendix 3). Funnel plots for each meta-analysis did not demonstrate any discernible asymmetry (Appendix 4).

### Obesity

For obesity, four articles met the inclusion criteria, all of which were suitable for pooling as all had defined obesity as BMI ≥ 30 kg/m^2^ and provided the RR for incident gout. All articles conducted multivariate analysis; two risk estimates were included from the article by Bhole et al. [15] which reported RRs separately for men and women.

**Fig. 2:**
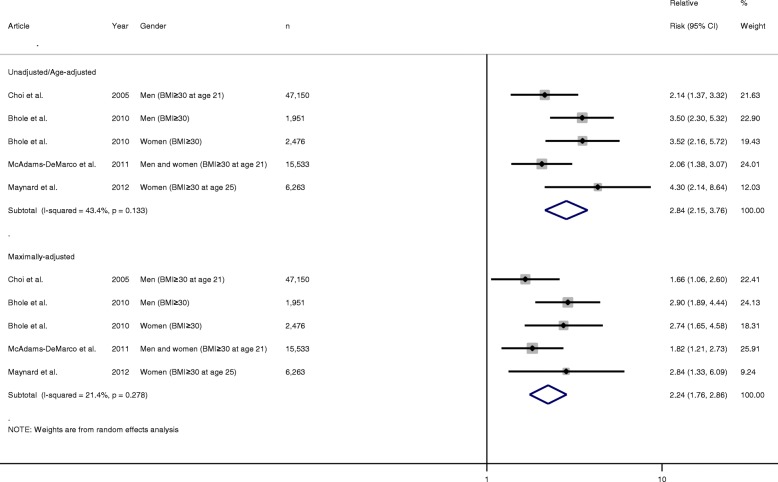
Forest plot showing pooled risk estimates for incident gout associated with body mass index ≥ 30 kg/m^2^. BMI body mass index, CI confidence interval

The pooled unadjusted/age-adjusted RR of incident gout in obese individuals compared with non-obese individuals was 2.84 (95% CI 2.15–3.76). The corresponding pooled multivariate-adjusted RR was 2.24 (1.76–2.86). There was no evidence of any statistically significant heterogeneity between the risk estimates as reflected by the low *I*^2^ values and non-significant *p* value (*I*^2^ = 21.4%, *p* = 0.278) (Fig. 2).

### Hypertension

**Fig. 3:**
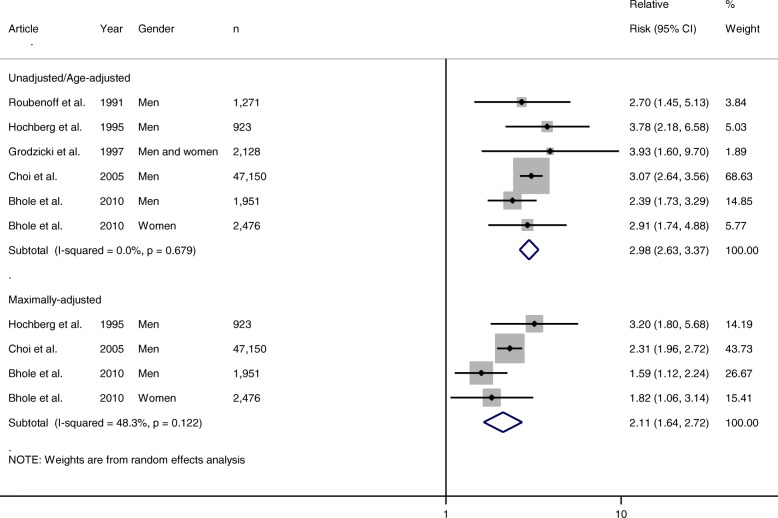
Forest plot showing pooled risk estimates (relative risk) for incident gout associated with hypertension. CI confidence interval

**Fig. 4:**
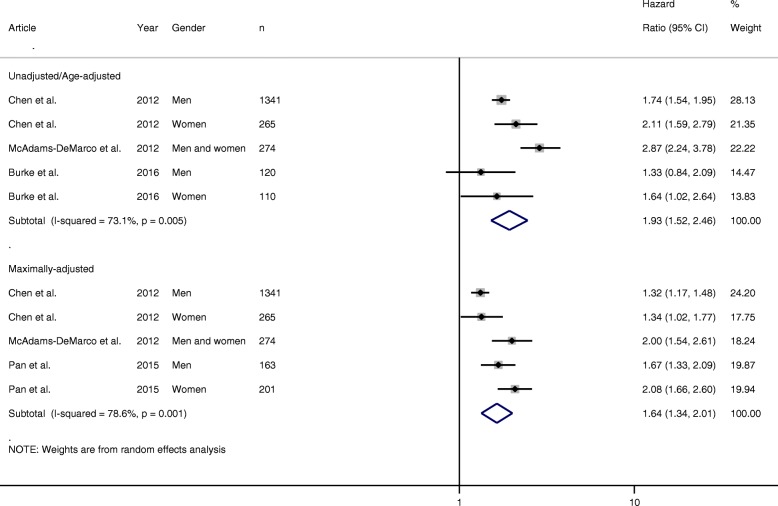
Forest plot showing pooled risk estimates (hazard ratios) for incident gout associated with hypertension. CI confidence interval

For hypertension, 10 articles met the inclusion criteria; of these, five provided RRs and four provided HRs which were suitable for pooling. For the RR meta-analysis, all five articles provided unadjusted/age-adjusted RRs, but only three articles provided multivariate-adjusted RRs. The pooled unadjusted/age-adjusted RR for incident gout in hypertensive individuals was almost three times higher than that in normotensive individuals (RR 2.98 (95% CI 2.63–3.37)). On pooling multivariate RRs, this risk was reduced, but remained significant (2.11 (1.64–2.72)). There was no evidence of any statistically significant heterogeneity between the risk estimates (*I*^2^ = 48.3%, *p* = 0.122) (Fig. 3). For the HR meta-analysis, three articles provided unadjusted/age-adjusted HRs [18, 23, 25] and three provided multivariate-adjusted HRs [18, 22, 25]. The pooled unadjusted/age-adjusted HR for incident gout in hypertensive individuals was almost two times higher than that in normotensive individuals (RR 1.93 (95% CI 1.52–2.46)). On pooling multivariate HRs, this risk was reduced, but remained significant (1.64 (1.34–2.01)). However, heterogeneity was reported as statistically significant (*I*^2^ = 78.6%, *p* = 0.001) (Fig. 4).

### Diuretic use

**Fig. 5:**
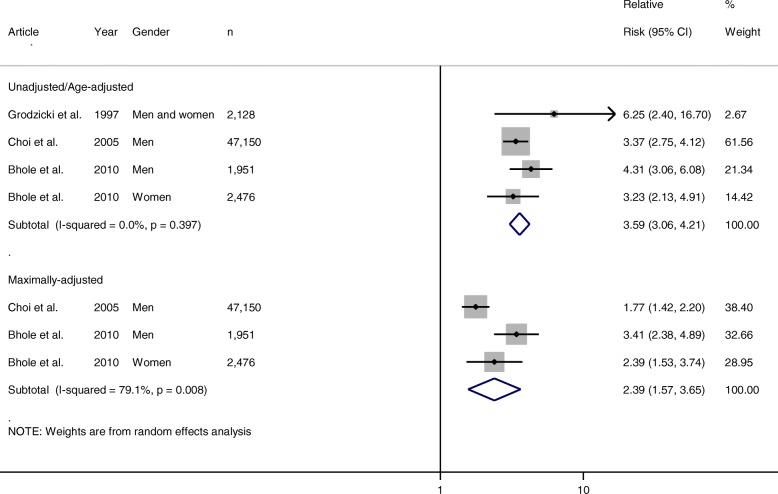
Forest plot showing pooled risk estimates for incident gout associated with diuretic use. CI confidence interval

Three of the six articles which met the inclusion criteria for diuretic use were suitable for inclusion in the meta-analysis. Only two studies provided multivariate-adjusted RRs suitable for pooling; however, these studies provided three relevant adjusted risk estimates. The pooled unadjusted/age-adjusted RR of incident gout in people taking diuretics compared to those not taking diuretics was 3.59 (95% CI 3.06–4.21). The corresponding pooled adjusted RR was 2.39 (1.57–3.65). Evidence for statistically significant heterogeneity was identified for the pooled multivariate-adjusted RRs (*I*^2^ = 79.1%, *p* = 0.008), but not for the unadjusted/age-adjusted RRs (*I*^2^ = 0.0%, *p* = 0.397) (Fig. 5).

## Discussion

This systematic review and meta-analysis of cohort studies has shown that in primary care and general populations, obesity, hypertension and diuretic use are all independent risk factors for incident gout. Each of these more than doubled the risk of developing gout.

Although this is the first meta-analysis of hypertension as a risk factor for gout, our findings for the other risk factors of obesity and diuretic use are supported by previous reviews. Our findings concerning obesity are consistent with those of a recent systematic review and meta-analysis which found that increasing BMI was a risk factor for the development of gout [26]. However, in contrast to our research, this meta-analysis included case–control studies as well as cohort studies, and included some studies of populations with hyperuricaemia, who are at greater risk of gout than the general population, perhaps explaining higher relative risks than those seen in our study. Hueskes et al. [8] published a systematic review examining the risk of gout associated with diuretics. They concluded that there was a trend that patients using either loop or thiazide diuretics were at an increased risk of gout; however, they reported that the magnitude and independence of this association in different studies was inconsistent and that evidence to support stopping diuretics in those with gout was lacking [8]. An important consideration is that their outcome was specifically defined as ‘acute gouty arthritis’ or ‘chronic tophaceous gout’, which is in contrast to the more inclusive outcome used in this study which was incident gout. This previous study did not attempt to pool risk estimates from different studies and was therefore unable to quantify the risk incurred by diuretic use. This systematic review also included randomised controlled trials, cohort studies and case–control studies, whereas our review included only cohort studies.

We have shown that obesity, hypertension and diuretic use are all important risk factors for incident gout. The prevalence of obesity is rising within the UK as well as globally and it has been linked to co-morbidities and mortality; as a result, the obesity epidemic has become a major public health concern. Previous research has demonstrated the benefits of weight reduction interventions in preventing gout [27] and our study has added further evidence to the need to tackle obesity due to its strong association with gout. Hypertension is primarily managed in primary care; careful selection of therapeutic agents can help to reduce the risk of future gout. This study also suggests that diuretics should be avoided in those at risk of developing gout, where possible, and alternatives considered.

Our study had a number of strengths including the comprehensive search strategy and literature review process, with no restrictions on language. By considering only primary care and population-based cohort studies for inclusion, we ensured that the results would be generalisable to most patients with gout, who are managed exclusively in primary care. We only included cohort studies in our review, reducing the effect of recall bias frequently encountered in case–control studies and allowing certainty of any temporal relationships between exposure and outcome [28]. Finally, as the risk estimates included in the meta-analyses were adjusted for the other risk factors of interest (i.e. obesity estimates were adjusted for hypertension, hypertension estimates were adjusted for diuretic use, etc.), we are confident these risk estimates are independent. As a result, it appears that hypertension is a risk factor for gout independent of diuretic use, but none of the included studies adjusted for other anti-hypertensive drugs which can cause hyperuricaemia. Therefore, we were unable to investigate whether the effect of hypertension was also independent of these.

Limitations in our work include that some studies had used specific samples (e.g. health professionals, university students), meaning their sampling frames with lower social deprivation are likely to underestimate the risk of incident gout. Other limitations include, firstly, that one-quarter of the articles did not specifically indicate that they had excluded individuals with a previous diagnosis of gout and, secondly, that variation may exist between pooled multivariate relative risks due to adjustments for different factors within different studies. However, regarding this latter point, several factors were the same (e.g. age, gender) and the majority of articles adjusted for the most important factors (in the case of this review, BMI, hypertension and/or diuretic use). In relation to this, although we are confident on the role of risk for each of these three variables, we are unable to address risk through different interactions of these, which would be clinically useful. Finally, diagnosis of gout was predominantly determined through self-report as no study required gout to be defined using the gold standard of crystal visualisation in the synovial fluid. This raises the possibility of misclassification; however, this approach is not unusual in large population/primary care-based epidemiological studies.

## Conclusion

Obesity, hypertension and diuretic use are all risk factors for incident gout, independent of one another and each more than doubling the risk of developing gout compared with those without these conditions. Such patients should be recognised by clinicians as being at greater risk of developing gout and provided with appropriate management and treatment options. Future research into interactions between these individual risk factors would expand our understanding of the epidemiology and pathophysiology of gout. As diuretic use in hypertensive patients is likely and a large proportion of such patients will be overweight, future research should consist of prospective studies which consider the interaction between co-morbidities and examine how certain clusters of co-morbidities influence the risk of developing gout, building on the work of Richette et al. [29].

## Figures and Tables

**Fig. 6:**
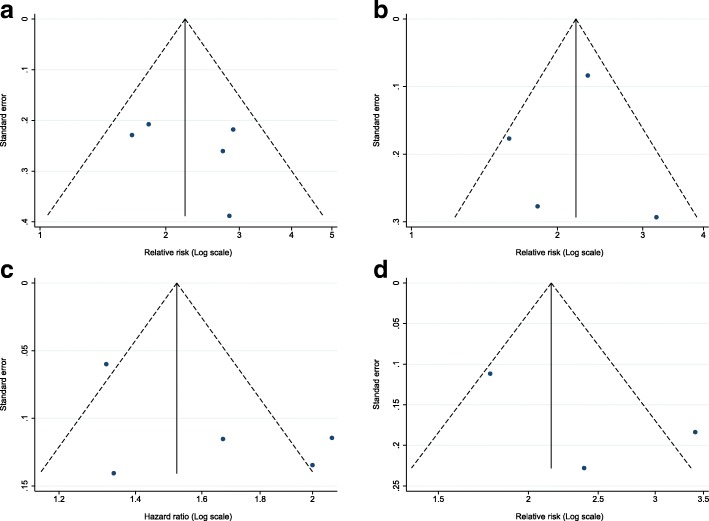
**a** Funnel plot of adjusted relative risk estimates for obesity. **b** Funnel plot of adjusted relative risk estimates for hypertension. **c** Funnel plot of adjusted hazard ratio estimates for hypertension. **d** Funnel plot of adjusted relative risk estimates for diuretic use

## Appendices

### Appendix 1

**Table 3:** Search Strategy

**Gout search terms**	
exp Gout/	
gout*.ti,ab.	
podagra.ti,ab.	
toph*.ti,ab.	
MeSH descriptor: [Gout] explode all trees *(Cochrane search only)*	
**Obesity search terms**	
exp Obesity/	
obes*.ti,ab.	
Body Mass Index/	
BMI.ti,ab.	
MeSH descriptor: [Obesity] explode all trees *(Cochrane search only)*	
MeSH descriptor: [Body Mass Index] this term only *(Cochrane search only)*	
**Hypertension search terms**	
exp Hypertension/	
hypertens*.ti,ab.	
(blood adj3 pressure).ti,ab.	
MeSH descriptor: [Hypertension] this term only (*Cochrane search only)*	
**Diuretics search terms**	
exp Diuretics/	
(loop adj3 diuretic*).ti,ab.	
(high-ceiling adj3 diuretic*).ti,ab.	
MeSH descriptor: [Diuretics] explode all trees *(Cochrane search only)*	

### Appendix 2

**Table 4:** Articles reviewed in full, but subsequently excluded (n=35)

Author	Year	Article title	Reason for exclusion
Ogryzlo	1960	The renal factor in the etiology of primary gout	Not cohort
Mertz & Schindera	1968	Secondary gout six years after acute renal failure	Not cohort
De Muckadall & Gyntelberg	1976	Occurrence of gout in Copenhagen males aged 40-59	Gout not an outcome
Seidell et al	1985	Fat distribution of overweight persons in relation to morbidity and subjective health	Gout not an outcome
Tsitlanadze et al	1987	Incidence and various risk factors for gout in the Georgian SSR	Not cohort
Van Noord et al	1990	The relationship between fat distribution and some chronic diseases in 11,825 women participating in the DOM-project	Gout not an outcome
Hoiberg & McNally	1991	Profiling overweight patients in the US Navy: Health conditions and costs	Based on RCT
Scott & Higgens	1992	Diuretic induced gout: A multifactorial condition	Not cohort
Youssef et al	1995	Does renal impairment protect from gout?	Not cohort
Gurwitz et al	1997	Thiazide diuretics and the initiation of anti-gout therapy	Not cohort
Lin et al	2000	Community based epidemiological study on hyperuricemia and gout in Kin-Hu, Kinmen	Not general population
Lin et al	2000	The interaction between uric acid level and other risk factors on the development of gout among asymptomatic hyperuricemic men in a prospective study	Not cohort
Takahashi et al	2000	Increased visceral fat accumulation in patients with primary gout	Not cohort
Lin et al	2006	Association of obesity and chronic disease in Taiwan	Not cohort
Miao et al	2008	Dietary and lifestyle changes associated with high prevalence of hyperuricemia and gout in the Shandong coastal cities of Eastern China	Not cohort
Zhu et al	2010	The serum urate-lowering impact of weight loss among men with a high cardiovascular risk profile: the Multiple Risk Factor Intervention Trial	Not cohort
Barskova et al	2011	Main factors of gender dimorphism of gout (estrogens and diuretics vs alcohol and genetics)	Not cohort
Chang	2011	Dietary intake and the risk of hyperuricemia, gout and chronic kidney disease in elderly Taiwanese men	Not cohort
Kawashima et al	2011	Association between asymptomatic hyperuricemia and new-onset chronic kidney disease in Japanese male workers: a long-term retrospective cohort study	Gout not an outcome
Primatesta et al	2011	Gout treatment and comorbidities: A retrospective cohort study in a large US managed care population	Gout not an outcome
Lin et al	2012	Prevalence of hyperuricemia and its association with antihypertensive treatment in hypertensive patients in Taiwan	Not cohort
Chen et al	2013	Impact of obesity and hypertriglyceridemia on gout development with or without hyperuricemia: A prospective study	Not cohort
Krishnan	2013	Chronic kidney disease and the risk of incident gout among middle-aged men: a seven-year prospective observational study	Not cohort
Lin et al	2013	The association of anthopometry indices with gout in Taiwanese men	Not cohort
McAdams-DeMarco et al	2013	A urate gene-by-diuretic interaction and gout risk in participants with hypertension: results from the ARIC study	Not cohort
Ozturk et al	2013	Demographic and clinical features of gout patients in Turkey: a multicenter study	Not general population
Wang et al	2013	Risk factors for gout developed from hyperuricemia in China: a five-year prospective cohort study	Not general population
Lu et al	2014	Contemporary epidemiology of gout and hyperuricemia in community elderly in Beijing	Gout not an outcome
Pan et al	2015	Bidirectional association between hypertension and gout: The Singapore chinese health study	Not cohort
Wang et al	2015	Chronic kidney disease as a risk factor for incident gout among men and women: retrospective cohort study using data from the Framingham Heart Study	Not general population
Abeles et al	2015	Hyperuricemia, gout, and cardiovascular disease: an update	Not cohort
Bao et al	2015	Lack of gene-diuretic interactions on the risk of incident gout: the Nurses' Health Study and Health Professionals Follow-up Study	Not general population
Jing et al	2015	Prevalence and correlates of gout in a large cohort of patients with chronic kidney disease: the German Chronic Kidney Disease (GCKD) study	Not general population
Dalbeth et al	2015	Body mass index modulates the relationship of sugar-sweetened beverage intake with serum urate concentrations and gout	Not cohort
Drivelegka et al	2016	Comorbidity pattern at the time of gout diagnosis: A population- and register-based case-control study from Western Sweden	Not cohort

### Appendix 3

**Table 5:** Quality appraisal scores of articles included in meta-analysis using the Newcastle-Ottawa Scale (NOS)

Article	Selection				Comparability	Outcome		
	1	2	3	4	1	1	2	3
	Is exposed cohort representative?	How was non-exposed cohort selected?	How was exposed cohort selected?	Clear, outcome wasn’t present?	Are cohorts compatible?	How was outcome assessed?	Was follow-up long enough?	Adequate cohort sample followed-up?
Roubenoff et al. 1991	C	A*	A*	B	A*, B*	B*	A*	B*
Hochberg et al. 1995	C	B	D	B	A*, B*	B*	A*	B*
Grodzicki et al. 1997	B*	A*	A*	B	-	B*	B	D
Choi et al. 2005	C	A*	C	A*	A*, B*	B*	A*	B*
Bhole et al. 2010	A*	A*	A*	A*	A*, B*	B*	A*	B*
McAdams-DeMarco et al. 2011	A*	A*	C	A*	A*, B*	C	A*	B*
Maynard et al. 2012	B*	A*	A*	A*	A*, B*	C	A*	B*
Chen et al. 2012	A*	A*	A*	A*	A*, B*	B*	A*	B*
McAdams-DeMarco et al. 2012	B*	A*	A*	A*	A*, B*	C	A*	B*
Pan et al. 2015	A*	A*	B*	A*	A*, B*	C	A*	D
Burke et al. 2016	B*	A*	A*	A*	A*, B*	C	A*	D

A indicates the highest methodological quality whereas D indicates the worst quality; An asterisk (*) denotes that the article has scored highest for that particular criterion. A comma (,) separating two scores denotes that an article i) matched exposed and non-exposed and ii) adjusted for potential confounding factors

## Abbreviations

ARIC: Atherosclerosis Risk In Communities
BMI: Body mass index
CI: Confidence interval
HR: Hazard ratio
MeSH: Medical subject headings
NOS: Newcastle–Ottawa Scale
OR: Odds ratio
RR: Risk ratio
ULT: Urate lowering therapies
